# Right ventricular dysfunction assessed by cardiovascular magnetic resonance is associated with poor outcome in patients undergoing transcatheter mitral valve repair

**DOI:** 10.1371/journal.pone.0245637

**Published:** 2021-01-29

**Authors:** Maximilian Spieker, Jonathan Marpert, Shazia Afzal, Athanasios Karathanos, Daniel Scheiber, Florian Bönner, Patrick Horn, Malte Kelm, Ralf Westenfeld

**Affiliations:** 1 Medical Faculty, Division of Cardiology, Pulmonology and Vascular Medicine, University Hospital Duesseldorf, Heinrich-Heine University Duesseldorf, Duesseldorf, Germany; 2 Cardiovascular Research Institute Duesseldorf, Medical Faculty, Heinrich-Heine University, Duesseldorf, Germany; Ospedale del Cuore G Pasquinucci Fondazione Toscana Gabriele Monasterio di Massa, ITALY

## Abstract

**Aims:**

To evaluate whether CMR-derived RV assessment can facilitate risk stratification among patients undergoing transcatheter mitral valve repair (TMVR).

**Background:**

In patients undergoing TMVR, only limited data exist regarding the role of RV function. Previous studies assessed the impact of pre-procedural RV dysfunction stating that RV failure may be associated with increased cardiovascular mortality after the procedure.

**Methods:**

Sixty-one patients underwent CMR, echocardiography and right heart catheterization prior TMVR. All-cause mortality and heart failure hospitalizations were assessed during 2-year follow-up.

**Results:**

According to RV ejection fraction (RVEF) <46%, 23 patients (38%) had pre-existing RV dysfunction. By measures of RV end-diastolic volume index (RVEDVi), 16 patients (26%) revealed RV dilatation. Nine patients (15%) revealed both. RV dysfunction was associated with increased right and left ventricular volumes as well as reduced left ventricular (LV) ejection fraction (all p<0.05). During follow-up, 15 patients (25%) died and additional 14 patients (23%) were admitted to hospital due to heart failure symptoms. RV dysfunction predicted all-cause mortality even after adjustment for LV function. Similarly, RVEDVi was a predictor of all-cause mortality even after adjustment for LVEDVi. Kaplan-Meier survival analysis unraveled that, among patients presenting with CMR indicative of both, RV dysfunction and dilatation, the majority (78%) experienced an adverse event during follow-up (p<0.001).

**Conclusion:**

In patients undergoing TMVR, pre-existing RV dysfunction and RV dilatation are associated with reduced survival, in progressive additive fashion. The assessment of RV volumes and function by CMR may aid in risk stratification prior TMVR in these high-risk patients.

## Background

Transcatheter mitral valve repair (TMVR) with the MitraClip system has evolved into an established treatment for patients with mitral regurgitation (MR) who are at elevated surgical risk. While peri-interventional mortality is low and the majority of patients clinically benefits from MitraClip implantation, 1-year mortality is largely determined by prognosis of underlying heart failure (HF) and comorbidities. Right ventricular (RV) dysfunction is an established prognosticator in patients with HF and after cardiac surgery, respectively [1,2].

In patients undergoing TMVR, only limited data exist regarding the role of RV function. Previous studies assessed the impact of pre-procedural RV dysfunction stating that RV failure may be associated with increased cardiovascular mortality after the procedure [3–5]. In contrast, Godino et al. indicated that RV dysfunction was not a predictor of mid-term clinical outcome [6]. Furthermore, the presence of concomitant tricuspid regurgitation (TR) is another known independent predictor of outcome in patients undergoing MitraClip implantation [7,8]. However, hitherto, no study assessed right heart function in patients receiving MitraClip by comprehensive cardiovascular magnetic resonance (CMR) imaging, the current gold standard for the assessment of myocardial volumes and function.

The aim of this study was to investigate the impact of pre-existing RV dysfunction assessed by CMR and right heart catheterization (RHC) on clinical outcomes after MitraClip procedure.

## Materials and methods

### Study population

Sixty-one patients undergoing MitraClip (Abbott Vascular, Santa Clara, California) implantation at the university hospital Duesseldorf, Germany were included between 2014–2019 and underwent CMR, echocardiography and RHC prior to TMVR. Patients enrolled had severe, degenerative or functional MR and were considered at elevated surgical risk by an interdisciplinary heart team. The study was approved by the ethics committee of the Heinrich-Heine University Duesseldorf (study number 6110R) and performed in accordance with the Declaration of Helsinki. All patients gave informed written consent.

We stratified patients according to the presence/absence of RV systolic dysfunction according to the RV ejection fraction (RVEF). Similarly, patients were separated into groups according to the presence/absence of RV dilatation. For assessment of RV dilatation, the RV end-diastolic volume index (RVEDVi) assessed by CMR was matched to age and gender specific reference values for each patient.(9) In this regard, in men <60 years, RVEDVi >111 ml/m^2^, and in men ≥60 years RVEDVi >101 ml/m^2^ was defined as RV dilatation. In women <60 years, RVEDVi >96 ml/m^2^, and in women ≥60 years RVEDVi >84 ml/m^2^ was defined as RV dilatation [9].

### Cardiovascular magnetic resonance imaging

CMR was conducted with a 1.5 Tesla MRI scanner (Achieva, Philips Medical Systems, Best, The Netherlands) using a 32-channel phased array coil. Functional and structural assessment was accomplished by cine steady state free precession images in standard long axis geometries (two-, three- and four-chamber view) as well as in short axis orientation with full ventricle coverage from basis to apex (slice thickness 8 mm, echo time 1.6 ms, repetition time 1.5 ms, FA = 60°, matrix size 184 x 2013 pixels, res = 8 × 1.4 × 1.4 mm^3^, 30 phases per cardiac cycle, breath-hold). Velocity encoded images for calculation of flows were acquired at ascending aorta and pulmonary trunk with a standard sequence. Pulmonary and aortic flow analysis was accomplished in 47 and 61 patients, respectively. In the remaining cases, echocardiography was used for determination of MR and TR severity. We used a commercial software (cmr42, Circle Cardiovascular Imaging Inc., Calgary, Alberta, Canada) for automatic delineation of ventricular borders in end-diastole and systole as well as calculation of volumes. Quality inspection was done manually. Left ventricular (LV) and RV end-diastolic volume and systolic volume and were matched to body surface area in order to calculate LV and RV systolic and end-diastolic volume indices (LVESVi/RVESVi/LVEDVi/RVEDVi). The LV and RV stroke volume index (LVSVi/RVSVi) was the difference between LVEDVi and LVESVi as well as RVEDVi and RVESVi. Ejection fraction (EF) was stroke volume divided by end-diastolic volume and expressed as a percentage. MR fraction was calculated by the difference between total LVSV minus total aortic forward flow, divided by total LVSV and multiplied with 100. Similarly, TR fraction was calculated as follows: [(TR fraction = total RVSV-total pulmonary forward flow)/total RVSV) × 100].

#### Right heart catheterization

RHC was performed at the time of coronary angiography, as part of a standardized protocol for the comprehensive assessment of MR severity and hemodynamic characterization. In summary, fluid-filled catheters connected to pressure transducers were used to determine pressures. After review of hemodynamic data, the following pressures were collected: mean right atrial (RA) pressure, systolic and end-diastolic pressures of the RV and the pulmonary artery (PA), as well as mean PA pressure, PA wedge pressure (PAWP). Cardiac output was assessed by the Fick method and indexed with body surface area to calculate cardiac index. Pulmonary vascular resistance and systemic vascular resistance were calculated as previously described.

### Follow-up

All-cause mortality and heart failure (HF) hospitalizations were assessed during 2-year follow-up. The clinical course was monitored by follow-up examinations, phone calls to the referring cardiologists and the patients`primary physicians or the patients themselves.

### Statistical analysis

All analyses were performed using Graphpad Prism 8 (Graphpad Software, San Diego, USA) and SigmaPlot (Systat Sotware Inc., San Jose, California, USA). Data for continuous variables are presented as mean ± SD or median with interquartile range (IQR). Categorical variables are presented as frequencies and proportions. Continuous variables were compared between two groups with independent-samples Student's t-test (normally distributed), Mann-Whitney U test (non-normally distributed), and chi-square test for categorical data. ROC analysis and Youden´s Index was used to calculate optimal cut-off values for RV systolic dysfunction (RVEF) and RV dilatation (RV dilatation). Correlations between continuous variables were assessed by Spearman’s rho. Unadjusted Cox proportional hazard model were performed to assess predictors of all-cause mortality and HF hospitalization. RV dysfunction was adjusted for LV ejection fraction, and RV dilatation was adjusted for LVEDVi. Multivariate analysis with more than two parameters was not performed due to the low number of events. The Kaplan-Meier method was used to determine the event-free rate. For all analyses, a p-value of <0.05 was considered to be statistically significant.

## Results

### Patient’s characteristics and CMR imaging

Baseline patient’s characteristics are shown in Table 1. Mean age was 78±9 years, 57% were female. Median NT-proBNP was 2440 (1226–4408) ng/l. The majority of patients had functional MR (74%), while one quarter of patients presented with degenerative MR (26%). LV ejection fraction was 52±14 and RVEF was 49±12 (Table 2). Fifteen patients (25%) presented with HF with reduced EF, 10 patients (16%) had HF with mid-range EF, and the majority (36 patients; 59%) had HF with preserved EF. ROC analysis regarding all-cause mortality demonstrated an optimal cut-off of RVEF <46% for RV systolic dysfunction (AUC 0.603; sensitivity 0.667; specificity 0.717), and a cut-off of RVEDVi >111 ml/m^2^ (for RV dilatation) (AUC 0.636; sensitivity 0.333; specificity 0.978). According to RVEF <46%, 23 patients (38%) had RV systolic dysfunction prior MitraClip procedure. According to RVEDVi, 16 patients (26%) revealed RV dilatation. Nine patients (15%) had both, RV dysfunction and RV dilatation. The remaining patients (N = 31, 51%) exhibited normal RV dimensions and systolic function. Patients with RV systolic dysfunction had higher logistic EuroSCORE (p<0.001) and lower estimated glomerular function (p = 0.007), while patients with RV dilatation more often presented with pre-existing atrial fibrillation (p = 0.004) (Table 1). In the whole cohort, 11 patients (18%) had concomitant moderate TR and 16 patients (26%) presented with severe TR. The majority of patients with RV dilatation (75%) presented with concomitant moderate/severe TR, while only 33% of patients without RV dilatation had moderate/severe TR (p = 0.007). In patients with and without RV systolic dysfunction, there was no difference in the presence of moderate/severe TR (57% vs. 37%; p = 0.185).

**Table 1:** Baseline patient characteristics.

Baseline and Clinical Characteristics	Overall N = 61	RVEF ≥46% N = 38	RVEF <46% N = 23	p-Value	No RV-Dilatation N = 45	RV-Dilatation N = 16	p-Value
Age (years)	78±9	76±9	80±8	0.125	77±9	80±8	0.207
BMI (kg/m^2^)	25±5	25±5	25±6	0.914	25±6	25±4	0.812
Women, (%)	35 (57)	23 (61)	12 (52)	0.523	26 (58)	9 (56)	0.969
Hypertension, n (%)	54 (88)	34 (89)	20 (87)	0.765	42 (93)	12 (75)	**0.048**
Diabetes mellitus, n (%)	12 (20)	8 (21)	4 (17)	0.727	10 (22)	2 (13)	0.401
Vascular disease, n (%)	10 (16)	6 (15)	4 (17)	0.870	8 (18)	2 (13)	0.624
Coronary artery disease, n (%)	44 (72)	26 (68)	18 (78)	0.406	34 (76)	10 (63)	0.317
Previous CABG, n (%)	14 (23)	8 (21)	6 (26)	0.650	10 (22)	4 (25)	0.821
Previous VS, n (%)	9 (15)	6 (16)	3 (13)	0.770	9 (18)	1 (6)	0.202
Atrial fibrillation, n (%)	39 (64)	22 (58)	17 (74)	0.207	24 (53)	15 (94)	**0.004**
Log EuroSCORE (%)	23±15	19±12	31±17	**<0.001**	22±14	27±17	0.272
NYHA III/IV, n (%)	47 (77)	28 (74)	19 (82)	0.423	34 (76)	13 (81)	0.642
DMR, n (%)	16 (26)	13 (34)	3 (13)	0.069	10 (22)	6 (37)	0.233
FMR, n (%)	45 (74)	25 (66)	20 (87)	0.069	35 (78)	10 (63)	0.233
Tricuspid regurgitation, n (%)				0.136			**<0.001**
None, n (%)	8 (13)	5 (13)	3 (13)		8 (18)	0 (0)	
Mild, n (%)	26 (43)	19 (50)	7 (30)		22 (49)	4 (25)	
Moderate, n (%)	11 (18)	7 (18)	4 (17)		9 (20)	2 (13)	
Severe, n (%)	16 (26)	7 (18)	9 (39)		6 (13)	10 (63)	
Serum Creatinine (mg/dl)	1.4±0.9	1.3±0.7	1.7±1.2	0.065	1.4±1.0	1.5±0.6	0.831
Estimated GFR (ml/min)	50±21	56±22	41±17	**0.007**	52±21	46±21	0.406
Hemoglobine (mg/dl)	12±2	12±2	11±2	0.211	12±2	12±2	0.381
NT-proBNP (pg/ml)	2440 (1226–4408)	1736 (725–2920)	3310 (1681–5578)	0.216	1953 (1148–3310)	3160 (1237–6502)	0.459

Abbreviations: BMI = Body mass index; CABG = Coronary artery bypass grafting; VS = Valve surgery; NYHA = New York Heart Classification; DMR = Degenerative mitral regurgitation; FMR = Functional mitral regurgitation; GFR = Glomerular filtration rate; NT-proBNP = NT-pro-Brain natriuretic peptide.

**Table 2:** Cardiac magnetic resonance parameters.

CMR parameter	Overall N = 61	RVEF ≥46% N = 38	RVEF <46% N = 23	p-Value	No RV-Dilatation N = 45	RV-Dilatation N = 16	p-Value
Left atrial area index (cm^2^/m^2^)	19±5	17±4	20±5	**0.016**	18±4	21±6	**0.007**
LV end-diastolic volume index (ml/m^2^)	90±28	83±24	101±31	**0.016**	85±29	104±22	**0.021**
LV systolic volume index (ml/m^2^)	49±33	42±32	60±31	**0.033**	45±35	59±24	0.148
LV stroke volume index (ml/m^2^)	44±11	46±12	42±9	0.126	44±12	45±8	0.670
LV ejection fraction (%)	52±14	56±13	44±13	**<0.001**	54±15	45±12	**0.047**
Mitral regurgitation fraction (%)	33±14	31±14	34±15	0.505	32±14	34±18	0.792
Aortic regurgitation fraction (%)	9±7	9±7	9±7	0.814	9±5	9±10	0.997
Right atrial area index (cm^2^/m^2^)	15±6	14±5	17±5	0.050	13±5	19±8	**<0.001**
RV end-diastolic volume index (ml/m^2^)	79±28	72±20	92±34	**0.007**	67±15	115±25	**<0.001**
RV systolic volume index (ml/m^2^)	42±23	32±11	59±27	**<0.001**	35±15	67±22	**<0.001**
RV stroke volume index (ml/m^2^)	38±12	41±10	31±11	**0.001**	34±9	48±12	**<0.001**
RV ejection fraction (%)	49±12	57±5	36±7	**<0.001**	51±11	43±12	**0.017**
Tricuspid regurgitation fraction (%)	22±14	24±14	16±14	0.934	20±13	34±13	**0.028**
Heart rate (bpm)	77±15	76±14	79±15	0.445	75±14	83±15	0.136
Cardiac index (ml/min/m^2^)	3.3±0.8	3.3±0.8	3.3±0.7	0.806	3.3±0.8	3.5±0.6	0.228

Abbreviations: LV = Left ventricular; RV = Right ventricular.

**Fig 1:**
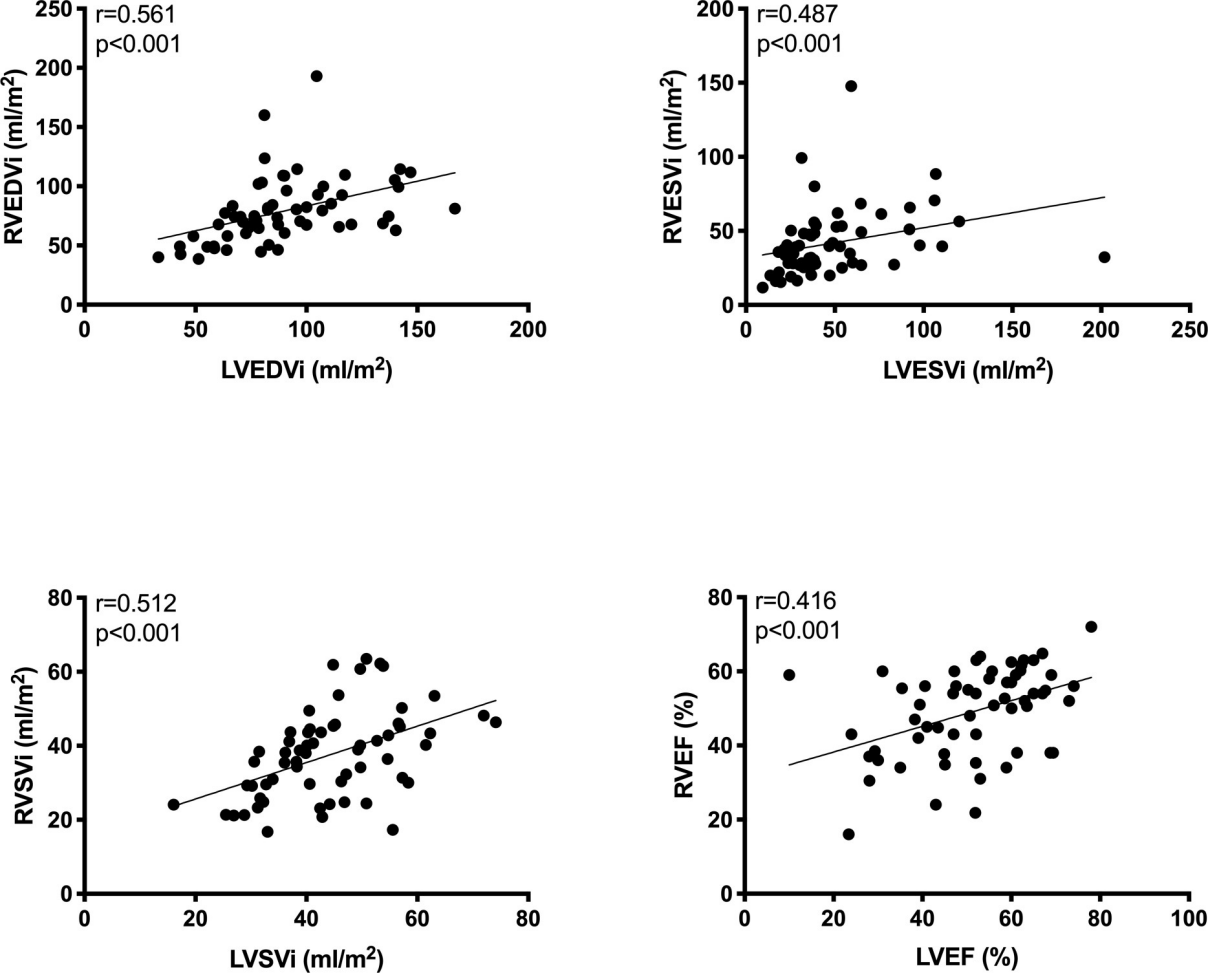
Relationship between cardiovascular magnetic resonance (CMR) right and left ventricular volumes (end-diastolic-, systolic- and stroke volume indices) and function (ejection fraction). Abbreviations: RVEDVi = Right ventricular end-diastolic volume index; LVEDVi = Left ventricular end-diastolic volume index; RVESVi = Right ventricular end-systolic volume index; LVESVi = Left ventricular end-systolic volume index; RVSVi = Right ventricular stroke volume index; LVSVi = Left ventricular stroke volume index; RVEF = Right ventricular ejection fraction; LVEF = Left ventricular ejection fraction.

CMR imaging showed increased right and left ventricular volumes as well as reduced LV ejection fraction in patients with RV dysfunction (all p<0.05) (Table 2). Patients with RV dilatation had elevated left atrial area index (p = 0.007) and LVEDVi (p = 0.021) as well reduced LV ejection fraction (p = 0.047) (Table 2). Together, there was a positive correlation between right and left ventricular volumes and function (Fig 1). Moreover, TR was more advanced in patients with RV dilatation (p = 0.028) (Table 2). S1 Table shows echocardiographic parameters of the study cohort according to the presence of RV dysfunction and RV dilatation.

**Fig 2:**
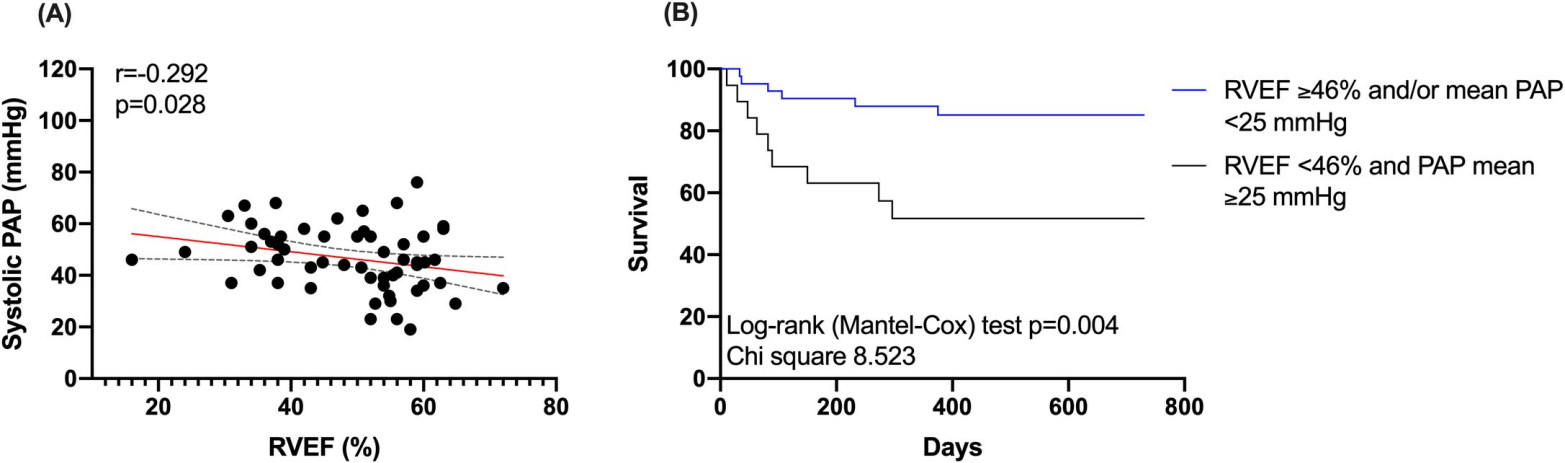
Relationship and prognostic relevance of right ventricular ejection fraction (RVEF) and pulmonary artery (PA) pressure. (A) The graph illustrates an inverse relationship between RVEF and systolic PA pressure. (B) The graph shows the Kaplan-Meier survival curves for all-cause mortality for patients with RVEF <46% and pulmonary hypertension (defined by mean PA pressure ≥25 mmHg) (red line) vs. patients with RVEF ≥46% and/or without pulmonary hypertension (blue line). Abbreviations: PA = Pulmonary artery; RVEF = Right ventricular ejection fraction.

RHC was performed in 56 patients (92%) prior MitraClip procedure and revealed a trend towards increased PA systolic pressure in patients with RV dysfunction (p = 0.058) (Table 3), while patients with RV dilatation had elevated diastolic RV pressure (p = 0.043) and mean RA pressure (p = 0.045) (Table 3). In addition, there was an inverse correlation between RVEF and systolic PA pressure (r = -0.292, p = 0.028) (Fig 2).

**Table 3:** Right heart catheterization data.

Right heart catheterization	Overall N = 61	RVEF ≥46% N = 38	RVEF <46% N = 23	p-Value	No RV-Dilatation N = 45	RV-Dilatation N = 16	p-Value
RA mean pressure (mmHg)	10±6	9±6	11±6	0.596	9±5	13±5	**0.045**
RV systolic pressure (mmHg)	48±15	46±17	51±10	0.210	48±16	47±12	0.902
RV diastolic pressure (mmHg)	9±6	8±5	9±5	0.598	8±5	11±5	**0.043**
PA systolic pressure (mmHg)	47±12	44±13	51±10	0.058	46±13	47±11	0.810
PA diastolic pressure (mmHg)	19±7	18±8	20±5	0.195	18±8	20±7	0.516
PA mean pressure (mmHg)	31±11	29±12	34±7	0.178	31±12	31±8	0.902
PAWP (mmHg)	22±11	22±9	24±9	0.487	21±9	26±6	0.145
SVR (dyn x sec x cm^-5^)	1963±726	2068±759	1823±654	0.306	1999±757	1843±596	0.583
PVR (dyn x sec x cm^-5^)	233±158	222±166	248±144	0.595	225±127	249±211	0.638
Cardiac index (ml/min/m^2^)	2.2±0.7	2.3±0.8	2.2±0.5	0.620	2.3±0.8	2.1±0.4	0.260

Abbreviations: RA = Right atrial; RV = Right ventricular; PA = Pulmonary artery; PAWP = Pulmonary artery wedge pressure; SVR = Systemic vascular resistance; PVR = Pulmonary vascular resistance; PAPi = Pulmonary artery pulsatility index.

### RV dysfunction and clinical outcome

Acute procedural success defined by MR grade ≤2 was achieved in 97%. Mean mitral valve pressure gradient assessed by echocardiography was 3.4±1.8 mmHg. Mean follow-up time was 581±174 days. During follow-up, 15 patients (25%) died, and additional 14 patients (23%) were admitted to hospital due to HF symptoms. Thus, 29 patients (48%) experienced an adverse event. In unadjusted Cox proportional hazard model, estimated glomerular filtration rate, diastolic RV pressure, mean RA pressure, LA area index, RA area index, RVEDVi, RVESVi, RV dysfunction and the combination of RV dilatation and RV dysfunction were predictors of all-cause mortality (Table 4). Even after adjustment for LV ejection fraction, RV dysfunction predicted all-cause mortality (HR 5.406 (1.691–17.277); p = 0.004). RV dilatation (according to age and gender matched cut-off values) was not associated with all-cause mortality after adjustment for LVEDVi (HR 2.238 (0.717–6.985); p = 0.165). However, RVEDVi (per ml/m^2^) predicted all-cause mortality even after adjustment for LVEDVi (HR 1.023 (1.007–1.039); p = 0.004). Regarding the combined endpoint, mean RA pressure, LA area index, RA area index, RVEDVi, RVESVi and the combination of RV dysfunction and RV dilatation predicted the occurrence of an adverse event during follow-up (Table 4).

**Table 4:** Unadjusted Cox proportional hazard model for all-cause mortality and the combination of all-cause mortality and heart failure hospitalization.

	All-cause Mortality			All-cause Mortality + HF Hospitalization		
	Hazard Ratio	95% CI	P-Value	Hazard Ratio	95% CI	P-Value
Estimated GFR (per ml/min/1.73m^2^)	0.962	0.936–0.988	**0.004**	0.987	0.970–1.005	0.166
Mean RAP (per mmHg)	1.175	1.054–1.309	**0.004**	1.093	1.017–1.173	**0.015**
Diastolic RVP (per mmHg)	1.108	1.000–1.226	**0.049**	1.068	0.997–1.145	0.060
PAWP (per mmHg)	1.047	0.994–1.104	0.085	1.013	0.973–1.053	0.537
LA Area Index (per cm^2^/m^2^)	1.097	1.024–1.176	**0.009**	1.063	1.003–1.126	**0.040**
RA Area Index (per cm^2^/m^2^)	1.079	1.014–1.148	**0.016**	1.062	1.010–1.116	**0.019**
RVEDVi (per ml/m^2^)	1.022	1.007–1.038	**0.003**	1.014	1.001–1.027	**0.040**
RVESVi (per ml/m^2^)	1.024	1.007–1.040	**0.005**	1.014	1.000–1.029	0.053
Tricuspid Regurgitation (per grade)	1.554	0.930–2.598	0.092	1.166	0.803–1.692	0.419
RV Dysfunction (RVEF <46%)	4.051	1.381–11.884	**0.011**	1.873	0.901–3.985	0.093
RV Dilatation (gender + age matched)	2.264	0.804–6.380	0.122	2.042	0.946–4.406	0.069
RV Dysfunction + RV Dilatation	6.160	2.156–17.604	**<0.001**	2.737	1.162–6.447	**0.021**

Abbreviations see Tables 2 and 3.

**Fig 3:**
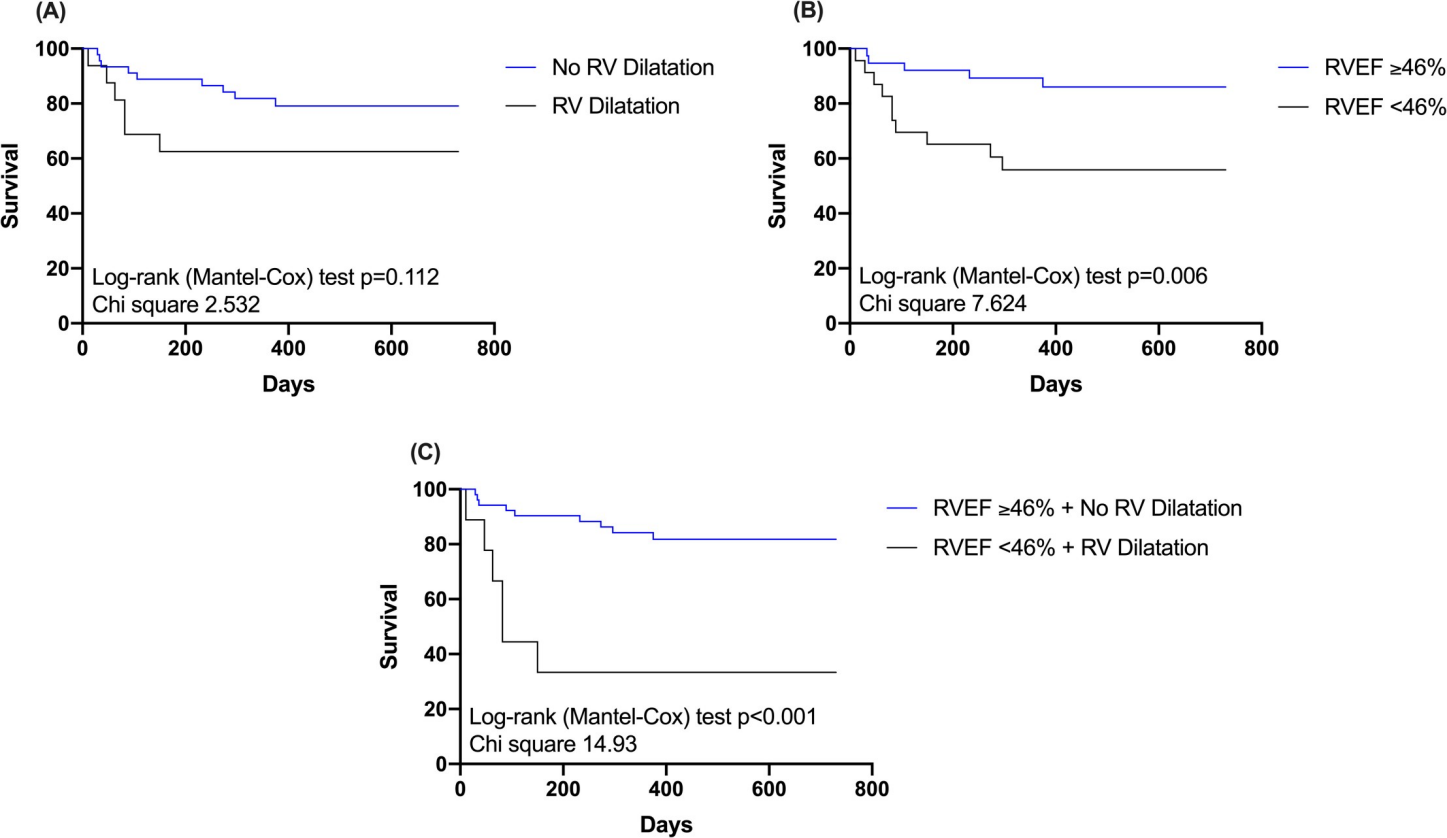
Kaplan-Meier survival curves for all-cause mortality according to cardiovascular magnetic resonance right ventricular volumes (end-diastolic volume) and function (ejection fraction). The figure displays Kaplan-Meier survival curves for (A) patients with right ventricular ejection fraction (RVEF) ≥46% and RVEF <46%; (B) patients with and without RV dilatation; and (C) patients presenting with both, RVEF <46% and RV dilatation. Abbreviations: RVEF = Right ventricular ejection fraction; RV = Right ventricular; PAP = Pulmonary artery pressure.

Kaplan-Meier survival analysis for all-cause mortality illustrates that patients with RV systolic dysfunction had increased all-cause mortality compared to those with preserved RV function (log-rank test p = 0.006) (Fig 3). Patients with RV dilatation showed numerically inferior survival compared to them without, however, without reaching statistical significance (log-rank test p = 0.112) (Fig 3). Six out of 9 patients (66%) who had both, RV systolic dysfunction and RV dilatation, died during follow-up (log-rank test p<0.001) (Fig 3).

**Fig 4:**
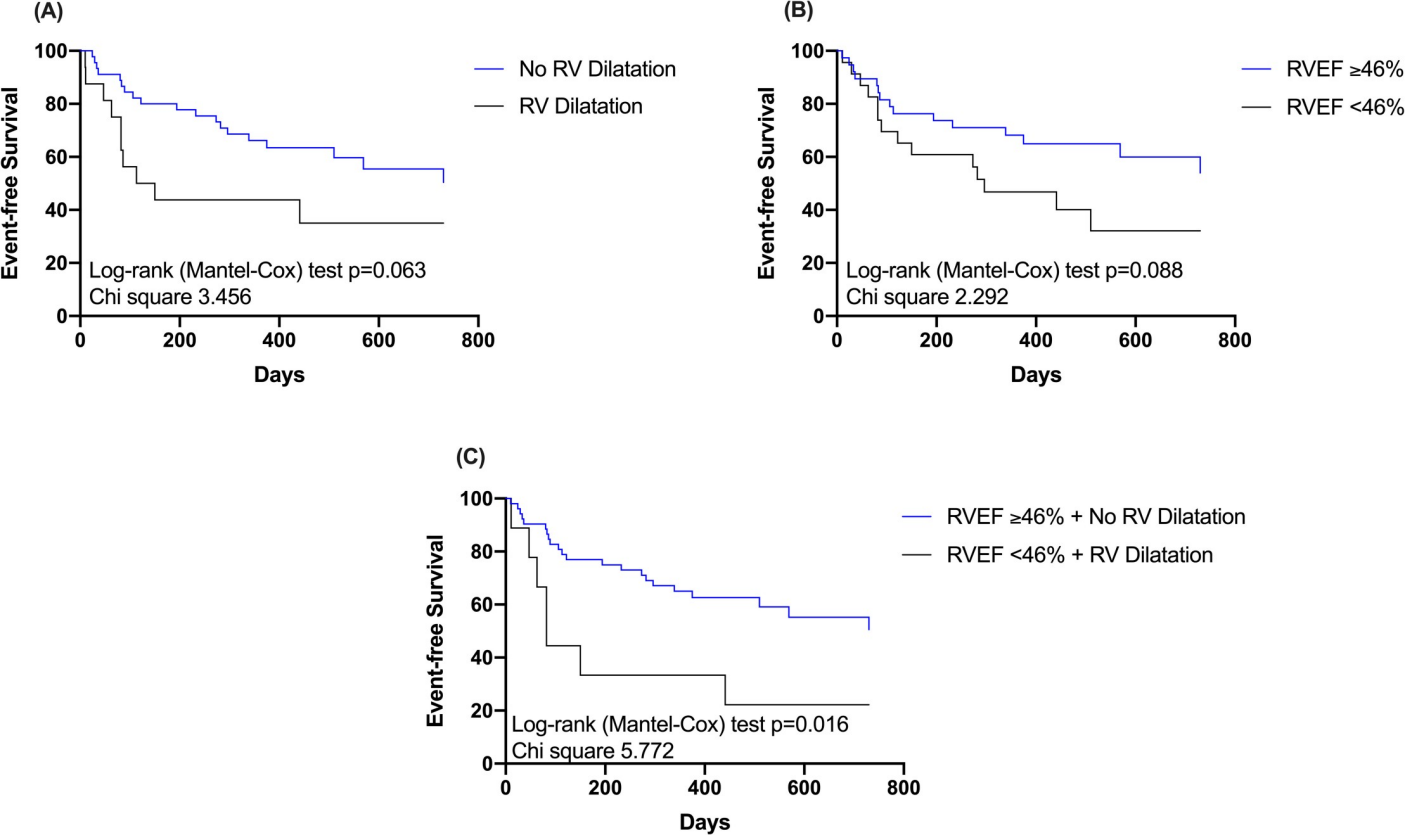
Kaplan-Meier survival curves for all-cause mortality and heart failure hospitalization according to cardiovascular magnetic resonance right ventricular volumes (end-diastolic volume) and function (ejection fraction). The figure displays Kaplan-Meier survival curves for (A) patients with right ventricular ejection fraction (RVEF) ≥46% and RVEF <46%; (B) patients with and without RV dilatation; and (C) patients presenting with both, RVEF <46% and RV dilatation. Abbreviations: RVEF = Right ventricular ejection fraction; RV = Right ventricular; PAP = Pulmonary artery pressure.

Regarding the combined endpoint, patients with RV systolic dysfunction (log-rank test p = 0.088) and patients with RV dilatation (log-rank test p = 0.063) tended to have an unfavorable outcome compared to those without (Fig 4). Among patients with both, RV systolic dysfunction and RV dilatation, 7 out of 9 patients (78%) experienced an adverse event during follow-up (log-rank test p<0.001) (Fig 4).

Eighteen patients had pulmonary hypertension (defined by mean PA pressure ≥25 mmHg) and RV systolic dysfunction (RVEF <46%). These patients had an all-cause mortality of 47% compared to 14% in patients without (log-rank test p = 0.004) (Fig 2).

## Discussion

In the current study, we evaluated the role of RV function and dimensions assessed by the current gold standard, CMR imaging, in patients undergoing TMVR with the MitraClip. Our study population represents a typical mixture of inoperable, high- and intermediate risk patients with symptomatic, severe MR that currently receive TMVR treatment in a real-world clinical setting. We demonstrate that pre-procedural RV systolic dysfunction and RV dilatation are associated with a poor prognosis, even following effective TMVR. The patient cohort presenting both, RV systolic dysfunction and RV dilatation, exhibit additive mortality. These findings suggest that the accurate assessment of RV function and dimensions may aid in risk stratification in patients undergoing MitraClip procedure.

### RV dysfunction and outcome

In our study, only 51% of patients had ordinary RV dimensions and function, while the majority of patients revealed RV systolic dysfunction (38%) or RV dilatation (26%). Nine patients (15%) presented with both. The presence of RV dysfunction was strongly associated with poor clinical outcome that fosters previous reports and underlines the importance of an accurate pre-procedural RV assessment. Overall, RV dysfunction is a known predictor of poor cardiovascular and overall outcome in different HF populations [1,10]. In patients with degenerative MR, RV dysfunction is associated with reduced survival, regardless of systolic LV function and surgical repair [11,12]. Moreover, in patients with HF and functional MR, RV dysfunction was an independent predictor of mortality [13].

The role of pre-procedural RV dysfunction in patients undergoing MitraClip procedure has not yet been clearly defined. Several reports using an echocardiographic assessment of RV function in this context reported conflicting results: Godino et al. stated that RV dysfunction (defined by tricuspid annular systolic excursion (TAPSE) <16 mm; RV peak systolic velocity doppler imaging (PSV_tdi_) <10 cm/s) was not associated with inferior outcome [6]. In contrast, other studies revealed prognostic importance of pre-existing RV dysfunction in patients undergoing MitraClip implantation. In this regard, Orban et al. showed that in a study population with biventricular HF, impaired RV function (assessed by TAPSE and visual assessment of RV function) and pulmonary hypertension were independent predictors of outcome [5]. Moreover, in the study of Kaneko et al. RV dysfunction (defined by TAPSE <15 mm) was associated with worse survival and LV dysfunction [3]. Giannini et al. demonstrated that patients with severe RV failure (assessed by PSV_tdi_) had an increased risk for cardiovascular mortality despite MitraClip treatment [4]. At least, Osteresch et al. demonstrated that patients with RV dysfunction (defined by TAPSE <16 mm) were less often responder to MitraClip treatment and showed an unfavorable long-term outcome [14]. Our results broaden these previous observations as we included patients in a real-world clinical setting and provided state-of-the-art RV assessment by CMR and RHC. In our cohort, patients with pre-procedural RVEF <46%, showed a higher risk of all-cause mortality and HF hospitalization during follow-up compared with patients with preserved RVEF (≥46%).

The reasons for RV dysfunction in MitraClip patients are multifactorial and include left HF with pressure overload from increased left-sided filling pressures and PA pressures transmitted to the right side; volume overload from fluid retention; pulmonary diseases; cardiomyopathies; and/or septal dysfunction that leads to ventricular interdependence [15]. In this regard, there was a correlation between left and right ventricular volumes and function, indicating an advanced disease severity in patients with RV dysfunction (Fig 1). Despite, RV dysfunction was a predictor of mortality even after adjustment for LV ejection fraction. Thus, RV dysfunction seems more sensitive than LV function for predicting worse outcomes. In addition, we observed an inverse relationship between RVEF and systolic PA pressure (Fig 3), that is in keeping with previous literature [16]. This could be related to the fact that an increased RV afterload further deteriorates RV performance. In this regard, pulmonary hypertension is an established predictor of adverse outcome in HF patients and is further known to increase the risk of death in cardiac surgery [17–19]. Previous literature in patients with biventricular HF undergoing MitraClip procedure demonstrated that patients with both, pulmonary hypertension and depressed RV function had a very high 1-year mortality of 77% [5]. Similarly, in our study cohort, patients with both, RV systolic dysfunction and pulmonary hypertension had an all-cause mortality of 47% compared to 14% in patients without (log-rank test p = 0.004). Therefore, the concomitant assessment of RV function and pulmonary hypertension may yield further important prognostic information.

However, not only RV function but also the assessment of RV dimensions may provide prognostic information. Generally, RV dilatation is considered to be a consequence of chronic volume and/ or pressure overload of the RV [15]. Furthermore, atrial fibrillation is known to be associated with RV chamber remodeling and dysfunction in heart failure patients [20]. In this regard, atrial fibrillation was present in the majority of patients with RV dilatation (94%). Elevated left atrial pressures caused by MR promotes left atrial remodeling with subsequent risk of atrial fibrillation. Elevations in left heart filling pressures, as commonly seen in atrial fibrillation, may adversely affect RV structure and function by increasing pulmonary pressures and pulsatile load to the RV, inducing pulmonary vascular disease, or both [21]. In addition, atrial dilatation further increase tricuspid annular diameter to worsen tricuspid regurgitation, which may further promote RV volume overload. Because of the greater compliance of the RV, it can accommodate larger increases in volume better than increases in pressure. A significantly dilated RV, however, might be a sign of advanced RV failure exceeding the adaptive stage [22]. In the current study, we provide an accurate assessment of RV dilatation with regards to current age- and gender matched, normal CMR values and show that RV dilatation was associated with an increased mortality rate and further HF hospitalizations during follow-up. Even after adjustment for LVEDVi, RVEDVi predicted all-cause mortality. But, not only dilation of the RV, but also increases in RA area were associated with adverse outcome (Table 4). Moreover, RV dilatation was associated with elevated RA and RV pressures. Thus, RV size better delineates the severity of pressure and/ or volume overload on the RV than ventricular function. Together, we emphasize that the assessment of RV dimensions should gain more recognition in the evaluation of the right heart in patients with severe MR.

### Advantages of CMR

Transthoracic echocardiography is widely available, fast and cheap, and therefore, the diagnostic approach of choice and performed on routine basis prior TMVR. Several studies investigated the role of RV dysfunction assessed by echocardiography in patients undergoing MitraClip implantation and reported conflicting results. To the best of our knowledge, the present study is the first to show that CMR assessment of RV function and volumes predicts outcome in patients undergoing MitraClip procedure. CMR offers several advantages: The capability to image in multiple planes and 3D volume acquisition lowers the need for geometric assumptions on RV shape [23]. Furthermore, high-spatial resolution enables distinguished discrimination between blood and endocardium. This seems of particular importance in the highly trabeculated RV to acquire precise systolic and end-diastolic measurements [24]. Furthermore, CMR offers the assessment of RV volumes and function with high accuracy and reproducibility that has been extensively validated [25]. Thus, the additional assessment of RV function by CMR imaging prior MitraClip procedure provides several advantages, and, moreover, facilitates prognostic estimation.

### Limitations

The current study has several limitations: First, we included a small but distinct patient cohort. However, this is the largest cohort that underwent CMR prior MitraClip procedure published so far. Our study population reflects a real-world setting with a typical mixture of inoperable, high- and intermediate risk patients that currently undergo MitraClip implantation. The majority of patients had functional MR (74%), thus, results cannot be directly transferred to patients with degenerative MR. Moreover, validation in larger cohort seems necessary. In this regard, multivariate analysis could not be performed due to the low number of events. Second, RVEF and RV dilatation are load-dependent and the presence of significant TR may influence both. Therefore, newer and more load independent measurements of RV function such as feature-tracking derived strain and strain-rate may provide a more precise measurement of RV function in future studies. Third, due to inclusion of patients with MRI-compatible pacemakers (only 5 out of 10), prevalence of patients with pacemakers represents 8% in our study cohort. Finally, we did not perform follow-up CMR imaging for assessment of RV function after MitraClip procedure, that might have yielded important information on the course of disease.

### Conclusion

An accurate assessment of RV function and RV dimensions is crucial in the screening process for TMVR as RV parameters provide important prognostic information that allow an estimation of HF severity and prognosis. In this regard, in our cohort with predominately functional MR patients, RV volumes and function were more sensitive variables than LV parameters for predicting adverse outcomes. Thus, a comprehensive RV assessment as can be performed by CMR in addition to echocardiography may aid in risk stratification prior TMVR.

## Supporting information

S1 Table Baseline echocardiographic parameters. (DOCX)

S1 Data Raw data. (PDF)

## Abbreviations

CMR: Cardiovascular magnetic resonance
EF: Ejection fraction
HF: Heart failure
LV: Left ventricle
MR: Mitral regurgitation
PA: Pulmonary artery
PAWP: Pulmonary artery wedge pressure
RA: Right atrium/atrial
RHC: Right heart catheterization
ROC: Receiver operating curve
RV: Right ventricle/ventricular
RVEDVi: Right ventricular end-diastolic volume index
RVEF: Right ventricular rejection fraction
TMVR: Transcatheter mitral valve repair
TR: Tricuspid regurgitation
