# Supplementary material for: Right ventricular dysfunction assessed by cardiovascular magnetic resonance is associated with poor outcome in patients undergoing transcatheter mitral valve repair
Source: PLoS One. 2021 Jan 29;16(1):e0245637. doi: 10.1371/journal.pone.0245637 (PMC7846001; doi:10.1371/journal.pone.0245637)
Supplement: S1 Table — (DOCX) [file pone.0245637.s001.docx]

**Table S1.** Baseline Echocardiographic Parameters.

| **Echocardiographic Parameters** | **Overall**  **N=61** | **RVEF 46%**  **N=38** | **RVEF <46%**  **N=23** | **p-Value** | **No RV-Dilatation**  **N=45** | **RV-Dilatation**  **N=16** | **p-Value** |
| --- | --- | --- | --- | --- | --- | --- | --- |
| LVEDD (mm) | 52±8 | 51±7 | 53±8 | 0.392 | 51±8 | 55±7 | 0.130 |
| LVEF (%) | 53+12 | 54±12 | 50±12 | 0.356 | 56±11 | 44±12 | **0.004** |
| MR ERO (cm^2^) | 0.24±0.15 | 0.21±0.09 | 0.25±0.20 | 0.111 | 0.24±0.17 | 0.25±0.10 | 0.736 |
| MR Vol (ml) | 43±22 | 41±17 | 45±28 | 0.093 | 43±25 | 42±17 | 0.958 |
| RVEDD (mm) | 34±11 | 33±9 | 37±12 | 0.207 | 32±9 | 40±12 | **0.013** |
| TAPSE (mm) | 18±4 | 19±4 | 17±4 | 0.284 | 19±4 | 17±4 | 0.187 |
| TR Grade, n (%) |  |  |  | 0.136 |  |  | **<0.001** |
| None, n (%) | 8 (13) | 5 (13) | 3 (13) |  | 8 (18) | 0 (0) |  |
| Mild, n (%) | 26 (43) | 19 (50) | 7 (30) |  | 22 (49) | 4 (25) |  |
| Moderate, n (%) | 11 (18) | 7 (18) | 4 (17) |  | 9 (20) | 2 (13) |  |
| Severe, n (%) | 16 (26) | 7 (18) | 9 (39) |  | 6 (13) | 10 (63) |  |
| TRPG (mmHg) | 49±12 | 47±15 | 51±12 | 0.466 | 47±14 | 56±8 | **0.049** |

Abbreviations: LVEDD=Left ventricular end-diastolic diameter; LVEF=Left ventricular ejection fraction; MR=Mitral Regurgitation; ERO=Effective regurgitant orifice; Vol=Volume; RVEDD=Right ventricular end-diastolic diameter; TAPSE=Tricuspid annular plane systolic excursion; TR=Tricuspid regurgitation; TRPG=Trans tricuspid pressure gradient.
