# Supplementary material for: Right ventricular dysfunction assessed by cardiovascular magnetic resonance is associated with poor outcome in patients undergoing transcatheter mitral valve repair
Source: PLoS One. 2021 Jan 29;16(1):e0245637. doi: 10.1371/journal.pone.0245637 (PMC7846001; doi:10.1371/journal.pone.0245637)
Supplement: S1 Data — (PDF) [file pone.0245637.s002.pdf]

Sex; Female=1	Age	BMI	BSA	Log EuroScore	NYHA
0	68	27,7	1,92	60,7	3
0	84	26,1	1,97	7,48	2
1	74	23,2	1,78	11	2
1	46	24,7	1,69	3,92	2
0	59	21,4	2,1	3	3
1	79	20,6	1,61	19	1
0	75	22,8	2,13	37,18	3
0	76	20	1,87	22	3
1	89	35,2	1,71	14	3
1	88	28	1,62	20,23	3
0	65	26,6	1,89	11	3
1	85	30,5	1,87	25	2
0	81	20,6	1,61	29,5	3
1	72	34,5	1,82	17	3
1	74	22,9	1,64	2	2
1	91	24,2	1,64	27,88	2
0	64	23,9	2,02	2,04	3
0	80	24,2	1,82	10,56	2
1	87	15,8	1,62	29	2
0	78	31,1	2,01	25	4
1	84	22,5	1,43	10	2
1	73	22,1	1,87	18	3
1	73	20,3	1,57	23,67	3
1	80	19	1,8	7,9	3
1	75	29,1	2,07	26	4
1	86	26,6	1,78	11,39	3
1	72	30,3	1,72	31,1	3
1	73	18,6	1,46	5,2	3
1	70	18,1	1,6	20	3
1	74	21,5	1,52	19,3	3
0	80	22,8	1,93	30	3
0	71	27,1	1,87	8,5	3
0	75	32	2,13	8,7	3
1	80	24,2	1,79	26,3	3
0	65	27,7	1,99	10,7	4
1	79	29,7	1,91	7,46	3
1	77	29,7	1,81	30,36	3
0	93	24,1	1,87	41	3
1	83	23,9	1,9	46	3
0	59	32,3	2,2	4,37	3
1	70	26,1	1,78	30	3
1	78	20,8	1,68	32,7	2
0	79	21,9	1,69	27,17	2
0	78	28,4	1,99	64,61	3
0	83	24	2,07	23,9	3
1	92	24,7	1,59	27	3
0	91	1,74	2,21	36,5	3
0	80	24,5	1,93	28,11	4
1	83	21	1,53	13,8	3
1	78	30	2,09	36,5	3

1	81	27,6	1,86	35	3
1	82	26,4	1,67	66	3
1	81	21,5	1,56	30	3
0	70	26,4	1,77	42	3
0	75	31,6	2,18	5,8	2
0	100	19,8	1,72	10	3
0	86	28,3	1,91	42,9	3
1	79	31,3	1,83	4,48	3
1	72	30,8	2,08	25	4
0	77	27	1,91	11	2
1	79	21,1	1,76	47	4

Diabetes	Hypertension	CAD	Vascular Disease	Atrial Fibrillation
1	1	1	1	0
0	1	1	1	1
0	1	1	0	0
0	0	0	0	0
0	1	1	0	1
0	1	1	0	1
0	1	1	0	1
0	0	1	0	0
0	1	1	0	0
1	1	1	0	1
0	1	1	0	0
1	1	1	0	0
0	1	1	0	0
1	1	0	0	1
0	1	1	0	1
0	1	0	0	1
0	1	0	0	0
0	1	1	0	1
0	1	0	0	1
1	1	1	1	0
0	0	0	0	0
0	1	1	0	1
0	1	0	0	1
0	1	0	0	1
1	1	1	1	1
0	1	1	0	1
1	1	1	0	1
0	1	0	0	0
0	1	0	0	0
0	1	1	0	0
0	1	1	0	1
0	1	1	0	0
0	1	0	0	1
0	1	1	0	0
1	0	1	1	1
0	1	1	0	1
0	1	0	1	1
0	1	1	0	1
0	1	0	0	1
1	1	1	0	0
1	1	1	0	0
0	1	1	0	1
0	1	1	0	0
0	1	1	1	1
0	1	1	0	0
0	1	1	0	1
0	1	0	0	1
0	1	1	1	1
0	1	1	1	0
0	1	0	0	1

0	1	1	0	0
0	1	1	1	1
0	1	1	0	1
0	0	1	0	1
0	1	1	0	1
0	1	0	0	1
0	0	1	0	1
1	1	1	0	1
0	1	0	0	1
0	0	1	0	1
1	1	1	0	1

Previous Valve Surgery	Previous CABG	Etiology; Functional MR=1	PC_mean
0	1	1	29
0	0	1	19
0	0	1	24
0	0	0	35
0	0	1	17
0	0	1	10
1	0	1	18
0	0	1	
0	0	0	11
0	0	1	
0	1	1	-
1	1	0	14
0	1	1	38
1	0	1	15
0	0	1	24
0	0	0	16
0	0	1	9
0	0	1	26
1	0	0	12
0	0	0	12
0	0	0	17
1	0	0	24
0	0	0	22
0	0	1	20
0	0	1	30
0	0	1	14
0	1	1	40
0	0	0	24
0	0	0	36
0	0	1	32
0	0	0	25
0	1	1	15
0	0	0	22
0	1	1	13
0	1	1	12
1	0	1	25
0	0	1	37
0	0	1	23
1	0	1	10
0	0	1	18
0	0	1	
0	0	1	14
0	0	1	26
0	1	1	14
1	1	1	28
0	0	1	20
1	0	1	20
0	0	1	
0	0	0	25
0	0	1	49

0	0	1	22
0	1	1	24
0	0	1	14
0	1	1	38
0	0	1	-
0	0	0	
0	1	1	23
0	0	1	24
0	0	1	24
0	1	1	31
0	0	0	22

PC_a	PC_v	PA_sys	PA_dias	PA_mean	RV_sys	RV_ed
32	33	68	29	42	63	17
20	30	39	17	27	39	13
25	36	55	21	34	51	4
50	33	44	20	30	42	12
19	21	57	23	35	54	12
11	11	23	9	14	23	1
32	33	39	13	24	39	8
11	14	35	15	19	38	3
		36	18	25	35	10
-	-		21	58	103	8
18	21	46	22	34	47	0
33	51		37	58	82	1
16	22	32	16	22	30	8
45	25	55	12	33		
19	16	41	14	22	40	0
13	13	23	9	15	27	7
27	37	58	25	39	58	15
13	10	29	7	17	31	3
15	11	37	11	22	43	1
22	24	46	18	29	46	5
11	9	19	4	10	22	3
24	29	44	20	29	40	13
21	26	52	21	32	52	6
48	49	55	22	37	55	14
22	27	34	6	0	34	6
40	38	76	41	54	71	20
23	40	45	14	30	45	10
49	44	65	29	44	69	18
35	46	59	19	36		
8	7	29	8	19	25	3
19	16	36	13	24	36	12
31	32	49	19	33	49	14
13	28	43	12	26	43	3
16	24	30	9	20	25	5
30	33	45	16	29	45	8
38	44	62	28	42	60	13
27	33	40	17	25	42	14
18	17	37	17	25	38	7
28	29	43	11	26	43	2
15	24	37	16	22	40	10
34	37	63	26	42	64	7
17	18	67	20	40	72	3
25	39	51	17	30	51	11
31	32	45	10	20	45	
29	35	43	14	28	39	6
		60	30	47	60	3
24	40	52	22	35	54	11
30	41	58	23	43	57	0

26	24	56	25	36	56	11
24	34	55	22	38	56	13
22	22	35	16	27	34	3
41	46	53	28	39	53	18
-	-	42	17	28	42	7
		49				
26	26	46	20	32	51	12
28	31	46	22	33	46	19
21	36	55	21	36	54	11
36	34	50	20	33	49	12
28	24	68	30	43	68	16

RV_bd	RA_a	RA_v	RA_mean	PVR	SVR	CO
7	17	16	13	335		4,06
0	17	16	12			
0	8	6	5	228	3336	3,5
8	25	24	22		503	10,2
6	15	13	10	302	-	3,95
0	8	5	3	65	1735	3,27
6	8	3	1	94	1314	5,18
0	3	1	1	196	1996	4,49
5	13	13	12			2,6
-1	21	15	11	-	-	-
10	14	13	12	409	2160	3,52
20	23	23	18	521	2301	2,9
3	12	11	5	121	1434	4,63
				127	3257	3,78
4	13	14	12	110	2110	4,32
0	9	8	6	81	709	5,87
3	20	16	15	351	2375	2,96
0	3	2	2	155	1731	3,1
1	11	8	6			
-3	8	7	5	276	2441	3,47
0	3	2	2	97	1821	4,96
6	11	11	9	145	2661	3,31
0	10	11	6	881		3,08
0	11	14	9			
0	9	7	5			
27	28	27	22	337	2023	3,08
0	9	8	6	158	3085	3,03
6	18	13	13	221	2182	2,9
	15	15	14	87		3,7
-2	5	6	3	151	2223	4,25
4	14	12	10	171		4,19
0	17	17	13	242		
0	7	7	2	264		
9	12	12	11	106	1036	6
3	10	10	8			
-6	23	19	17	128	3400	2,49
0	17	15	14	92	1724	4,36
0	15	16	13	297	1269	4,03
0	2	2	1	91	1198	5,28
0	11	12	9	130		4,94
0	5	6	3	348	2088	3,68
1	5	6	2	548	1488	4,09
0	17	17	14	23		7,02
-9	3	3	1		2451	3,2
0				162		3,96
6	20	19	17		1897	4,55
0	15	13	7	354	3747	2,26
8	12	13	9	243	2078	4,6

0	12	10	9	290	1516	3,86
0	20	19	16	269	1535	4,17
0	13	15	11	261	1627	3,98
5	26	23	20	159	-	2,82
2	-	-	-	461	1120	4,86
4	17	13	11	241	1874	2,99
11	25	28	22	39	873	4,68
0	14	15	11	327	2344	2,93
2	17	17	12	34	1543	4,72
5	18	17	16	433	2350	3,88

CI	RA/PAWP	PAPi	LVEDD_CMR	LVESD_CMR	IVS_CMR
2,12	0,44827586	3	47,6	37,7	14,6
	0,63157895	1,83333333	52	29	9
1,97	0,20833333	6,8	62,9	36,9	16,2
6,1	0,62857143	1,09090909	51	48	8,6
1,95	0,58823529	3,4	57	43	10
1,98	0,3	4,66666667	53,7	42,6	11,7
2,41	0,05555556	26	61	35	18
			64	55	9
2,64	0,09090909	20	31	22	20
1,63		1,5	46,1	24,4	12,4
-		-1,9090909	55	43,1	17
1,88	0,85714286	2	52,3	33,1	16,3
1,77	0,47368421	-2,0555556	54	38	11,7
2,5	0,33333333	3,2	54,1	32,8	11,9
2,31			51	32,1	11,9
2,57	0,75	2,25	48	24,1	12,3
2,91	0,66666667	2,33333333	54	32	8,9
1,62	0,57692308	2,2	59,6	38,2	17,8
1,99	0,16666667	11	53,7	29,3	16
	0,5	4,33333333	60	43	11
2,43	0,29411765	5,6	48	28	10
2,55	0,08333333	7,5	56,3	35,6	7,3
1,97	0,40909091	2,66666667	50,9	43,5	10,9
1,71	0,3	5,16666667	55	28	55
	0,3	3,66666667	55	31	9
	0,35714286	5,6	52	33	10
1,85	0,55	1,59090909	48	31	13
2,08	0,25	5,16666667	46,8	31,9	10,3
1,81	0,36111111	2,76923077	63,2	43,7	8,2
2,4	0,4375	2,85714286	54	37	7
2,2	0,12	7	46,4	31,3	11
2,26	0,66666667	2,3	53	25	9
	0,59090909	2,30769231	62	43	7
	0,15384615	15,5	50	42,6	12,4
3,1	0,91666667	1,90909091	49	38	7
	0,32	3,625	46	39	10
1,38	0,45945946	2	62,3	49,3	11,3
2,33	0,60869565	1,64285714	68,7	50,2	11,7
2,04	1,3	1,53846154	56	38	15
2,4	0,05555556		63	58	12
			60,4	40,6	10,6
2,87	0,64285714	2,33333333	47,3	32,1	10,5
2,1	0,11538462	12,3333333	73,4	65,2	5,8
2,05	0,14285714	23,5	56	49	10
3,38	0,5	2,42857143	56,7	40,7	12,7
1,87	0,05	35	45,4	36,8	14,9
2,1			50,2	35,8	12,8
2,31		1,76470588	56	48	15
1,48	0,28	4,28571429	50,4	32,8	8,6
2,2	0,18367347	3,88888889	58	53	15

2,13	0,40909091	3,44444444	54	9,3	33,3
2,5	0,66666667	2,0625	61	50	14
2,47	0,78571429	1,72727273	49	33	10
1,59	0,52631579	1,25	60	52	8
2,23			65,3	46,9	10
			52	36	12,6
1,59	0,47826087	2,36363636	58	56	10
2,55	0,91666667	1,09090909	52,5	46,8	9,9
1,47	0,45833333	3,09090909	61	51,7	12,3
2,47	0,38709677	2,5	63,1	52,8	14,2
2,19	0,72727273	2,375	55	42,2	16,2

RVEDD_CMR	LVEDVi	LVESVi	LVSVi	CO_CMR	CI_CMR	LVEF_CMR
29,9	114,7395833	60,05208333	54,63541667	6,3	3,28125	47,6
38	84,7715736	27,9187817	56,852792	6,6	3,35025381	67
41,4	83,08988764	36,79775281	46,29213483	7,7	4,3258427	55,7
36,2	100	39,0532544	61,538462	8,23	4,86982249	61
45	97,14285714	58,57142857	38,19047619	5,2	2,47619048	39,4
33,9	79,50310559	47,20496894	32,23602484	4,6	2,85714286	40,6
42	67,6056338	18,3098592	49,295775	7,3	3,42723005	73
44	120,3208556	83,42245989	36,89839572	7	3,74331551	31
38	43,2748538	9,356725146	33,91812865	4,8	2,80701754	78
36,2	33,33333333	16,66666667	16,04938272	2,44	1,50617284	47,21
41,6	137,037037	64,92063492	71,95767196	7,2	3,80952381	53
32,7	49,09090909	18,5026738	30,58823529	4,9	2,62032086	62,3
42	87,26708075	32,48447205	54,7826087	5,2	3,22981366	62,8
60,5	66,75824176	21,59340659	45,16483516	5,3	2,91208791	67,7
38,3	78,47560976	201,8292683	47,19512195	5,3	3,23170732	60,1
43,2	100,0609756	25,91463415	74,14634146	7,9	4,81707317	74,1
34	64,35643564	23,76237624	40,59405941	6,2	3,06930693	63
48,4	77,47252747	36,81318681	40,65934066	4,88	2,68131868	52,14
	87,16049383	28,7654321	58,39506173	6,5	4,01234568	67
50	95,52238806	38,30845771	57,21393035	9,07	4,51243781	60
41	89,51048951	35,66433566	53,84615385	4,9	3,42657343	60
47,7	116,0427807	52,94117647	63,10160428	7	3,74331551	55
37,8	79,93630573	39,42675159	40,50955414	3,9	2,48407643	50,7
43	90	36,66666667	53,33333333	7,7	4,27777778	59
43	63,28502415	23,1884058	40,09661836	6,2	2,99516908	63
39	64,04494382	25,2808989	38,764045	4,8	2,69662921	61
25	90,11627907	54,06976744	36,04651163	4,2	2,44186047	10
34,3	51,43835616	19,52054795	31,23287671	3,4	2,32876712	62,1
48,8	111,1875	48,875	62,3125	8,3	5,1875	56,1
35	86,84210526	30,26315789	56,57894737	5,3	3,48684211	65
59,5	78,34196891	32,5388601	45,80310881	6,1	3,16062176	58,5
47	76,47058824	26,73796791	49,73262032	6,3	3,36898396	65
40	76,5258216	36,61971831	39,90610329	6,6	3,09859155	52
42,5	60,33519553	22,01117318	38,26815642	3,7	2,06703911	63,5
32	71,35678392	35,678392	36,180905	8,1	4,07035176	50,3
39	42,93193717	13,6125654	29,319372	4,4	2,30366492	69
45,7	105,1933702	64,86187845	40,27624309	6,4	3,5359116	38,3
60	142,2459893	91,97860963	50,80213904	7,55	4,03743316	35,4
41	82,63157895	38,42105263	44,21052632	5,9	3,10526316	53
46	134,5454545	110,4545455	32,72727273	5,7	2,59090909	24
44,2	107,1348315	51,57303371	55,56179775	8,7	4,88764045	51,9
46,1	73,92857143	23,0952381	50,83333333	5,3	3,1547619	68,8
34,3	166,9822485	120,0591716	46,86390533	4,8	2,84023669	28,1
53	110,5527638	68,3417085	42,211055	6,9	3,46733668	38,1
55,5	72,75362319	29,90338164	42,85024155	8	3,8647343	58,9
23,2	58,49056604	33,01886792	25,47169811	4,3	2,70440252	43,5
44,9	55,24886878	26,42533937	28,82352941	3,6	1,62895928	52,1
42	141,4507772	92,2279793	49,740933	5,9	3,05699482	35
57,3	82,61437908	25,29411765	57,32026144	6,8	4,44444444	69,4
42,3	77,03349282	46,93779904	30,19138756	6	2,8708134	39,1

	140,3225806	97,84946237	42,47311828	5,11	2,74731183	30
50	91,01796407	53,89221557	37,1257485	4,2	2,51497006	41
30	58,33333333	31,41025641	26,92307692	3,7	2,37179487	47
31	146,8926554	106,2146893	41,24293785	6,3	3,55932203	28
61,4	81,19266055	38,53211009	42,66055046	9,3	4,26605505	52
70	104,6511628	59,30232558	44,86046512	6,17	3,5872093	43
60	139,7905759	106,806283	32,984293	7	3,66492147	23,4
58,6	81,03825137	31,36612022	49,72677596	6,9	3,7704918	61,3
49,8	107,6442308	76,15384615	31,49038462	5,2	2,5	29,3
53,4	95,81151832	50,83769634	44,81675393	7,5	3,92670157	46,9
44,1	117,3863636	64,71590909	52,72727273	6,5	3,69318182	44,9

RVEDVi	RVESVi	RVSVi	RVEF_CMR	NTproBNP
65,625	28,64583333	36,45833333	56	2236
84,26395939	39,08629442	45,17766497	54	440
50,56179775	20,2247191	30,33707865	60	2861
67,4556213	27,81065089	40,23668639	59	1749
70,47619048	34,76190476	35,71428571	51	1723
44,72049689	19,8757764	24,8447205	56	927
74,17840376	35,68075117	38,96713615	52	302
67,9144385	27,27272727	41,17647059	60	2711
42,69005848	11,69590643	30,99415205	72	603
40,12345679	16,04938272	24,07407407	60	3630
74,6031746	26,98412698	48,14814815	64	3221
57,86096257	22,13903743	35,72192513	61,7	1420
67,70186335	25,46583851	42,85714286	63	1158
83,51648352	37,74725275	45,76923077	54,8	1943
64,63414634	32,31707317	32,31707317	50	325
82,31707317	35,97560976	46,34146341	56	3698
57,92079208	28,21782178	29,7029703	52	351
70,87912088	26,37362637	44,50549451	63	1418
46,41975309	16,35802469	30,0617284	64,8	1226
80,59701493	30,34825871	50,24875622	62,5	7859
109,0909091	47,55244755	61,53846154	57	539
92,51336898	39,57219251	53,47593583	58	598
103,1847134	53,69426752	49,49044586	48	20282
108,8888889	46,66666667	62,22222222	57	2652
77,29468599	37,19806763	40,09661836	52	766
46,06741573	19,1011236	38,76404494	59	1863
60,46511628	25	35,46511628	59	1388
38,69863014	15,4109589	23,28767123	60,2	2581
83,125	41,9375	43,375	50,8	1692
73,68421053	26,97368421	46,05263158	63	35000
101,9170984	48,1865285	53,67875648	52,7	433
74,86631016	34,75935829	40,10695187	54	437
69,95305164	31,92488263	38,02816901	54	2440
67,87709497	33,51955307	34,3575419	50,6	789
69,84924623	31,65829146	38,19095477	55	6607
49,21465969	19,89528796	29,31937173	59	2816
92,81767956	49,17127072	43,64640884	47	4408
114,4919786	51,01604278	63,47593583	55,4	3096
80	55,78947368	24,21052632	31	3019
68,63636364	39,54545455	29,54545455	43	1137
79,43820225	62,13483146	17,30337079	21,8	1713
64,88095238	40,47619048	24,4047619	38	413
81,18343195	56,39053254	24,73372781	30,5	5270
97,98994975	65,82914573	32,66331658	33	8034
60,38647343	40,09661836	20,77294686	34	3321
47,67295597	26,35220126	21,3836478	44,8	3299
48,86877828	28,05429864	21,26696833	43	2077
99,48186528	65,80310881	34,19689119	34	35000
81,69934641	50,32679739	31,37254902	38	1311
68,89952153	39,71291866	29,18660287	42	2350

62,90322581	40,32258065	23,11827957	36	3812
96,40718563	53,29341317	43,71257485	45	1237
49,35897436	28,20512821	21,15384615	43	5195
111,8644068	70,62146893	40,6779661	37	8298
123,6697248	80,04587156	43,62385321	35,3	3160
193,0232558	147,6744186	45,34883721	24	6502
105,2356021	88,48167539	16,7539267	16	4529
160,0546448	99,28961749	60,76502732	38	17936
99,85576923	61,44230769	38,41346154	38,5	4861
114,5549738	69,54	44,46	39	1584
109,7727273	68,40909091	41,36363636	37,7	-

Serum Creatinine	estimated GFR	Hemoglobine
1,3	58	11
1,2	57	11,2
0,7	84	15,4
3,3	16	12,6
0,93	90	14,1
1	56	13
1,2	63	11,2
1,1	64	5,8
1,1	46	13,2
1,3	36	10,8
1,42	51	14,5
1,1	46	10,5
1,6	40	11
1,2	46	12,4
0,8	69	12,2
0,83	62	12,8
1,4	54	11,9
1	71	14,2
0,93	55	14,6
2,8	21	8,4
0,6	86	12,3
0,8	71	11,9
2,2	21	12,9
0,7	83	15,7
1	55	10,5
1,1	48	10,7
1,4	39	10
1,4	38	11,7
0,7	83	13,6
3,5	12	8,8
1	70	12,8
1,1	73	14,9
0,7	94	11,5
1,3	40	12
0,7	97	9,4
1,3	39	12,4
0,9	60	12,5
1,7	34	10,8
1,2	44	9,5
1,6	46	13,1
0,5	98	12,3
1,1	51	9,9
1,3	52	12,4
2,74	21	10,4
1,3	52	10,4
1,6	27	11,8
1,7	34	13,7
6,7	10	8,3
1,1	48	13,6
0,9	66	12,1

0,9	31	8,8
1,4	34	10
1,2	44	9,3
1,5	46	12,7
1,51	45	10,6
2,3	23	10,5
1,4	45	14,6
1,35	37	9,6
1,2	45	15,3
2,03	30	10,4
3,2	13	11,1
